# Exploring the role of body mass index in relationship of serum nitric oxide and advanced glycation end products in apparently healthy subjects

**DOI:** 10.1371/journal.pone.0213307

**Published:** 2019-03-11

**Authors:** Elaheh Foroumandi, Mohammad Alizadeh, Sorayya Kheirouri, Mohammad Asghari Jafarabadi

**Affiliations:** 1 Department of Nutrition, Faculty of Nutrition and Food Sciences, Tabriz University of Medical Sciences, Tabriz, Iran; 2 Students’ Research Committee, Tabriz University of Medical Sciences, Tabriz, Iran; 3 Nutrition Research Center, Tabriz University of Medical Sciences, Tabriz, Iran; 4 Road Traffic Injury Research Center, Tabriz University of Medical Sciences, Tabriz, Iran; Fu Jen Catholic University, TAIWAN

## Abstract

This study aimed to identify any association of serum nitric oxide (NO) and advanced glycation end products (AGEs) with body mass index (BMI) in apparently healthy subjects. In this cross-sectional study, participants were 90 apparently healthy subjects, categorized into three BMI groups as follows: BMI≤19.5 (n = 21), 19.6≤BMI≤24.9 (n = 35), and BMI≥25 (n = 34). Serum levels of NO were measured by griess reaction method. Determination of serum pentosidine and carboxymethyllysine (CML) was done using ELISA. Median (95% confidence interval [CI]: lower- upper) of serum NO in subjects with BMI≥25 were 68.94 (CI: 55.01–70.56) μmol/L, which was higher compared with 19.6≤BMI≤24.9 and BMI≤19.5 groups (22.65 (CI: 19.29–28.17) μmol/L and 8.00 (CI: 9.12–29.58) μmol/L, respectively). Serum NO positively correlated with BMI in total subjects (r = 0.585, p<0.001), which this correlation was significant in both male and female groups (r = 0.735, p<0.001 and r = 0.476, p = 0.001, respectively). Serum pentosidine and CML were significantly lower in subjects with higher BMI. Further, BMI showed negative correlations with pentosidine and CML (r = -0.363, p<0.001 and r = -0.484, p<0.001, respectively). There were not any significant differences in serum NO, pentosidine, and CML levels between sex groups. After adjusting the effects of confounders (BMI, sex, age, and waist to hip ratio), serum NO significantly correlated with serum pentosidine and CML (r = -0.319, p = 0.003 and r = -0.433, p<0.001, respectively). It is concluded that higher BMI is accompanied by increased serum NO and suppressed pentosidine and CML.

## Introduction

Obesity is a multifactorial disorder which defined as an excessive fat accumulation that may impair health [1]. According to the World Health Organization in 2016, 39% and 13% of adults aged 18 years and over in the world were overweight and obese, respectively [2]. The obesity is a main risk factor for cardiovascular diseases, musculoskeletal disorders, diabetes mellitus and some cancers [3, 4]. Obesity is associated with inflammation due to the activation of pro-inflammatory signaling pathways, and expression of tumor-necrosis factor (TNF-alpha) in adipose tissue. There is evidence that obesity is associated with chronic inflammation, endothelial cell dysfunction and increased oxidative stress, which may affect nitric oxide (NO) production and activation [5].

Nitric oxide is produced through the oxidation of L-arginine in different cells from three isozymes of nitric oxide synthase (NOS) including eNOS (endothelial NOS), nNOS (neuronal NOS), and iNOS (inducible NOS). Nitric oxide, a gaseous molecule with a very short half-life, is involved in wide range of biological processes including vasodilatation of endothelium, oxidation of low-density lipoproteins, and inhibition of smooth muscle cell proliferation. Bioavailability of NO, as a physiologic regulator, depends on its generation and degradation [6]. Diminished bioavailability of NO in the body, is associated with cardiovascular diseases (CVD), hyperlipidemia (HLP), diabetes, obesity, and metabolic syndrome [7, 8].

Recent studies on obese subjects have shown that, raised concentrations of oxidized LDL and TNF-α may lead to down-regulation of eNOS expression via reduction in its mRNA half-life [9, 10]. On the other hand, obesity induced cytokines production, such as TNF-α that can blunt transportation of L-arginine into the endothelial cell which further contributes to diminished NO production [11]. The proinflammatory state in obesity leads to the production of NO, which in turn has the potential to mediate DNA damage through the generation of reactive nitrogen species (RNS) [12]. Interestingly, NO acts as a scavenger of many free radicals and, consequently suppresses dicarbonyl compounds’ formation, as intermediates in the formation of advanced glycation end products (AGEs) [13].

High formation of AGEs, accelerates in diverse conditions such as diabetes, coronary artery disease and aging [14]. AGEs’ production is a non-enzymatic process which is contributed to reaction of the carbonyl group of carbohydrates with free amino groups of proteins, nucleic acids or lipids [15]. Pentosidine and CML are the most commonly studied AGEs [16, 17]. Hyperlipidemia, hyperglycemia and oxidative stress are key contributors in the complex pathways leading to AGEs’ formation. As these metabolic conditions are also important characteristic features of obesity [18], it is expected that these compounds might accumulate under conditions of obesity.

There is some evidence indicating that excess accumulation of AGEs in the body may adversely affect endothelium cells that can reduce the half-life of mRNA which is involved in NOS expression, and consequently NO production [19, 20]. In an in vitro study, it was shown that expression and release of NO by eNOS was markedly reduced after exposure of cultured endothelial cells to AGE-modified proteins [21]. Therefore, we hypothesized that higher BMI may alter serum NO and AGEs levels. By considering globally increasing prevalence of obesity, we aimed to measure (1) levels of serum NO, pentosidine and CML with regard to BMI levels; to identify any association of serum NO, pentosidine and CML with anthropometric indices in apparently healthy subjects, and (3) to evaluate any correlation between serum NO and AGEs.

## Materials and methods

### Participants

This cross sectional study included 45 men and 45 women aged > 20 < y who were recruited by convenience sampling from the people who attended to public health centers affiliated to the Tabriz University of Medical Sciences, Iran from January to March 2016. People who were suffering from chronic diseases or mental illness, with regular drug therapy and history of smoking and alcohol consumption were excluded from the study. They all signed an informed consent. The protocol was approved by the ethical committee of Tabriz University of Medical Sciences, Tabriz, Iran (reference number: TBZMED.REC.1394.1032).

### Anthropometric measurements

Height and weight of participants were obtained using standardized techniques and instruments. The body mass index (BMI) was calculated as the weight in kilograms divided by the square of the height in meters [weight (kg)/Height (m^2^)]. The subjects were divided into three BMI groups as follows: BMI≤19.5, 19.6≤BMI≤24.9, and BMI≥25. Waist circumference (WC) was measured at the midpoint between the lower rib margin and the iliac crest. Hip circumference was measured at the level of maximum circumference of the buttocks. Waist to hip ratio (WHR) was calculated as WC divided by hip circumference [22].

### Blood sampling

Peripheral venous blood samples were obtained after 12–14 hours fasting from each subject and centrifuged 10 min at 300 ×g and separated serums were then stored at -20°C for analysis.

Serum NO was measured using the Griess reaction method [23]. In brief, serum samples were deproteinized by adding 500 μL absolute ethanol, followed by centrifugation at 10,000 ×g for 10 min. 100 μL of supernatant was applied to micro-plate well, and following addition of 100 μL vanadium (III) chloride (8 mg/ml), Griess reagents [50 μL sulfanilamide (2%) and 50 μL N-(1-Naphthyl) ethylendiamine dihydrochloride (0.1%)] were done to each well. After incubation at 37°C for 45 min, the absorbance was measured using a spectrophotometer at 540 nm.

### Determination of serum pentosidine and CML

Serum pentosidine and CML levels were detected using ELISA kits (Cat. No. E0004Hu and E1413Hu; Bioassay Technology Laboratory, Shanghai, China, respectively) following the manufacturer’s instructions. In brief, 50 μL of standards and 40 μL of the samples were added to the appropriate wells of a pre-coated plate together with the secondary antibody labelled with biotin. After an hour of incubation at 37°C, the wells were washed five times with wash buffer, chromogen solution was added and the mixture was incubated for 10 minutes at 37°C. Color development was then stopped, and the absorbance of the wells was measured at 450 nm.

### Statistical analysis

Data analyses were done using SPSS software (SPSS Inc., Chicago, IL, USA; Version 23). The normality of all variables’ distribution was checked by descriptive measures including coefficients of skewness and kurtosis, mean and standard deviation [24]. The NO, pentosidine, and CML values were log transformed to improve the normality of the distributions. Descriptive statistics were shown as mean± standard error of measurement (SEM) or Median (95% confidence interval [CI]: lower- upper). Analysis of covariance (ANCOVA) was used to analyze differences between serum NO, pentosidine and CML in study BMI and sex groups and LSD test applied for multiple comparisons. Partial correlation analysis was performed for determining probable correlations among continuous variables by considering confounders.

## Results

A total of 90 (n = 45 for each sex group) apparently healthy subjects were recruited in current study. The characteristics of the participants based on the gender and BMI are shown in Table 1. The mean± SEM age of total participants was 44.82±2.11. The mean of BMI in three BMI groups (BMI≤19.5, 19.6≤BMI≤24.9, and BMI≥25) were 18.22±0.37, 22.72±0.25 and 29.32±0.41 kg/m^2^, respectively. There was a significant difference between WHR of sex groups (p<0.05).

**Table 1:** Demographic characteristics and dietary intake of study participants.

Variables		BMI≤19.5 (n = 21)	19.6≤BMI≤24.9 (n = 35)	BMI≥25 (n = 34)	Total	
					Female** (n = 45)	Male** (n = 45)
Age (year)		51.00±5.60	40.71±3.21	45.23±2.80	44.89±3.14	44.75±2.85
Gender*	Female	9(20.0)	15(33.3)	21(46.7)	-	-
	Male	12(26.7)	20(44.4)	13(28.9)	-	-
BW (Kg)		42.38±1.59^A^^B^	64.90±2.17^A^^B^	87.42±2.33^A^^B^	70.42±3.21	65.89±3.09
BMI(kg/m2)		18.22±0.37^A^^B^	22.72±0.25^A^^B^	29.32±0.41^A^^B^	24.81±0.80	23.52±0.61
WC(cm)		46.57±1.66^A^^B^	77.68±2.68^A^^B^	103.29±2.36^A^^B^	84.69±3.92	75.51±3.61
WHR		0.88±0.02^a^^b^	0.88±0.01	0.94±0.02^a^^b^	0.88±0.01^c^^d^	0.92±0.01^c^^d^

Based on one way ANOVA analysis

BW: Body weight/ BMI: Body mass index/WC: Waist circumference/WHR: Waist to hip ratio

All data by mean± SEM

* n(percent)

**Based on Independent-samples T-test

^a-d^ Different lowercase letters represent difference (p<0.05) between study groups.

^A,B^ Different uppercase letters represent difference (p<0.001) between study groups.

**Fig 1:**
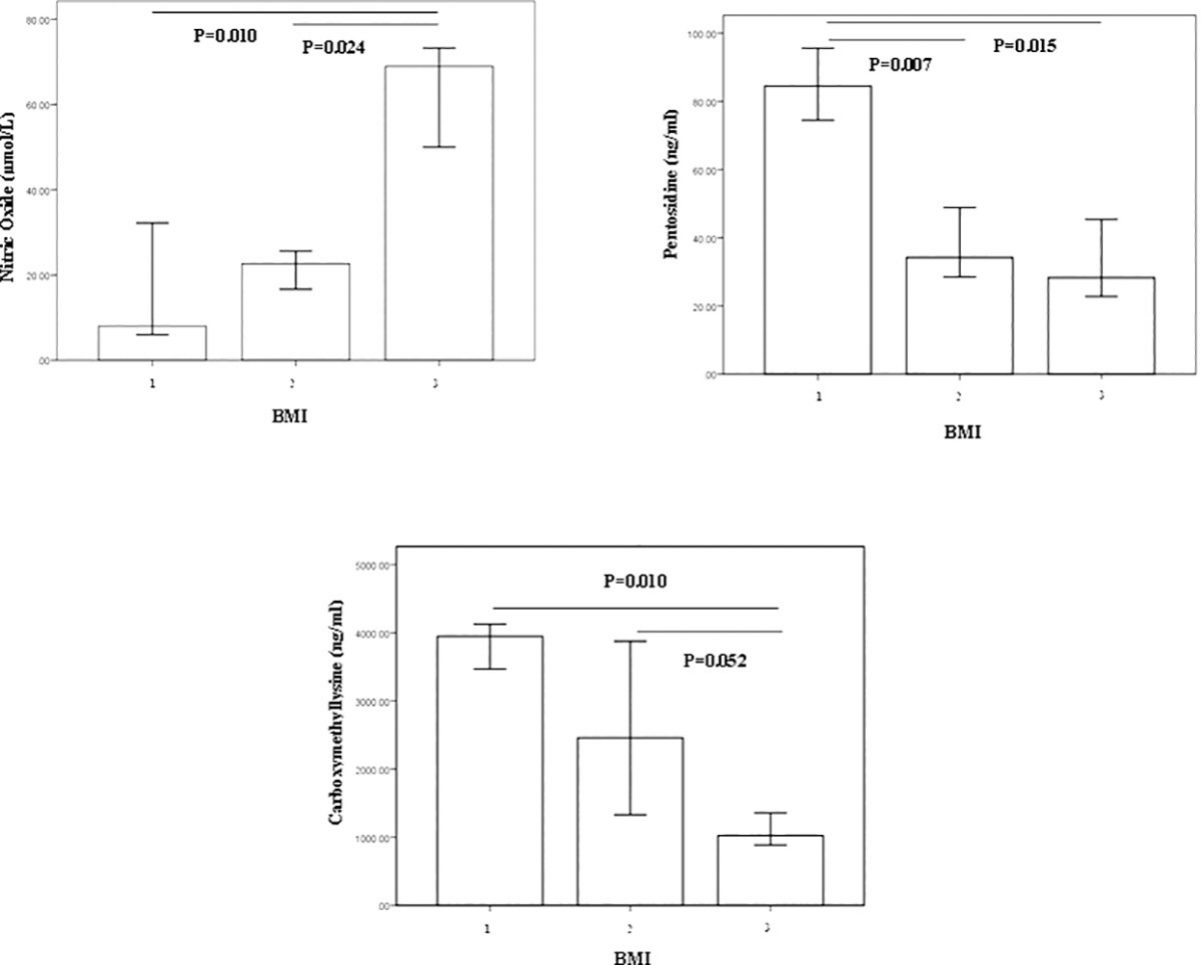
Bar plots representing concentration of serum nitric oxide, pentosidine, and carboxymethyllysine in BMI groups. BMI≤19.5 (1), 19.6≤BMI≤24.9 (2) and BMI≥25 (3). Lower and upper bar boundaries are lower and upper 95% coefficient intervals (CI), the upper line of the bar is median. Each bar plot reports comparison test p-values (ANCOVA) using log-transformed NO, pentosidine, and CML by adjusting confounder effects of age, sex, and WHR.

As shown in Fig 1, there was a downward trend in serum NO levels by BMI falling after adjusting the effects of sex, WHR, and age, which the median (95% [CI]: lower- upper) was 68.94 (55.01–70.56) μmol/L in the participants with BMI≥25, 22.65 (19.29–28.17) μmol/L in 19.6≤BMI≤24.9 group and 8.00 (9.12–29.58) μmol/L in BMI≤19.5 group. As seen in Fig 1, serum pentosidine and CML were higher in the participants with BMI≤19.5 compared with those with BMI≥25 (p<0.05). Median (95% [CI]: lower- upper) of serum pentosidine levels in BMI≤19.5, 19.6≤BMI≤24.9, and BMI≥25 groups were 84.50 (CI: 76.55–90.52), 34.22 (CI: 35.63–56.30), and 28.31 (CI: 30.89–47.81) ng/ml, respectively. The serum CML concentration (ng/ml) was 3945.70 (CI: 3203.85–4042.42), 2456.80 (CI: 2113.31–3104.24), and 1024.45 (CI: 1120.37–1818.71) in lower, normal and high BMI groups, respectively.

**Fig 2:**
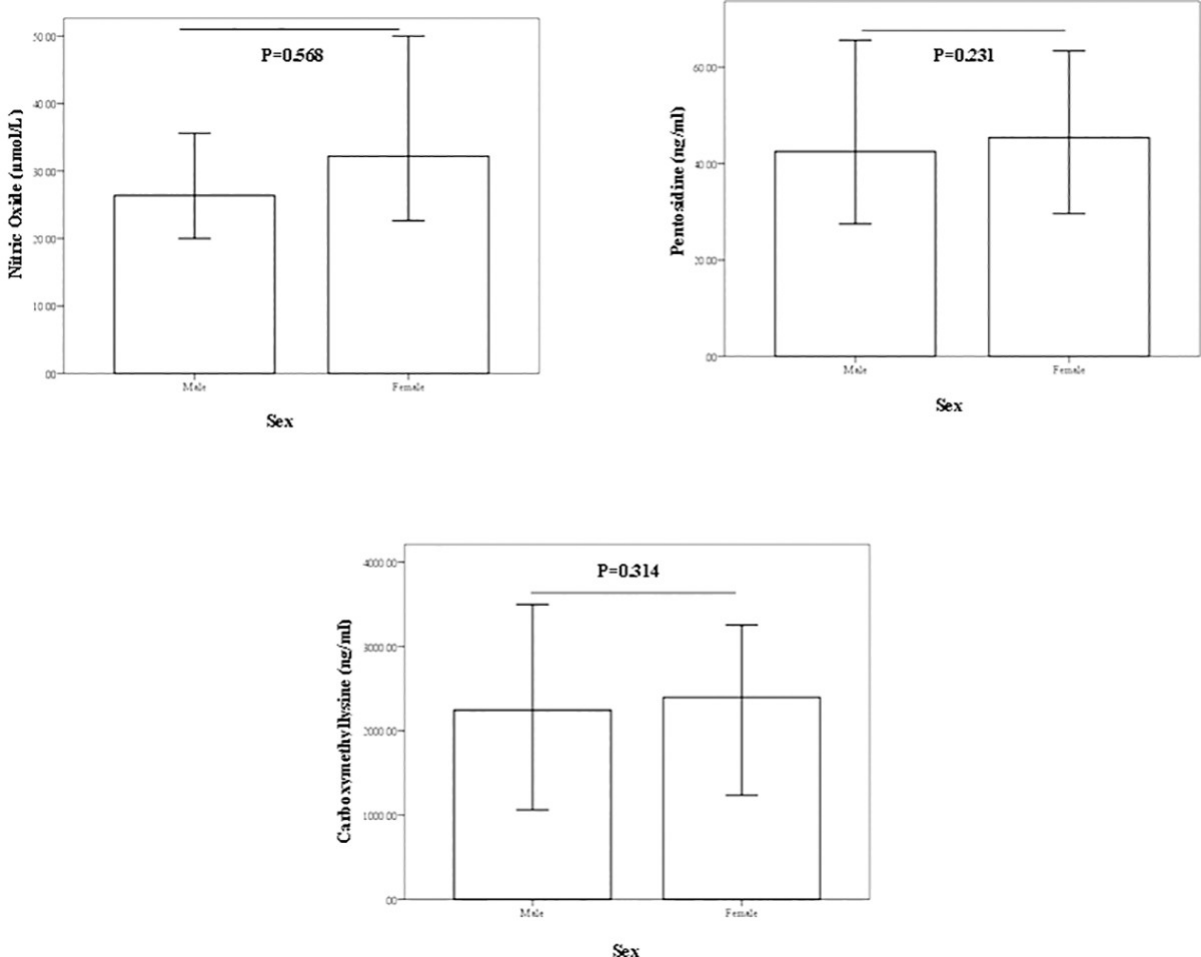
Bar plots representing concentration of serum nitric oxide, pentosidine, and carboxymethyllysine in each sex groups. Lower and upper bar boundaries are lower and upper 95% coefficient intervals (CI), the upper line of the bar is median. Each bar plot reports a comparison test p-value (ANCOVA) using log-transformed NO, pentosidine, and CML by adjusting confounder effects of age, BMI, and WHR.

**Fig 3:**
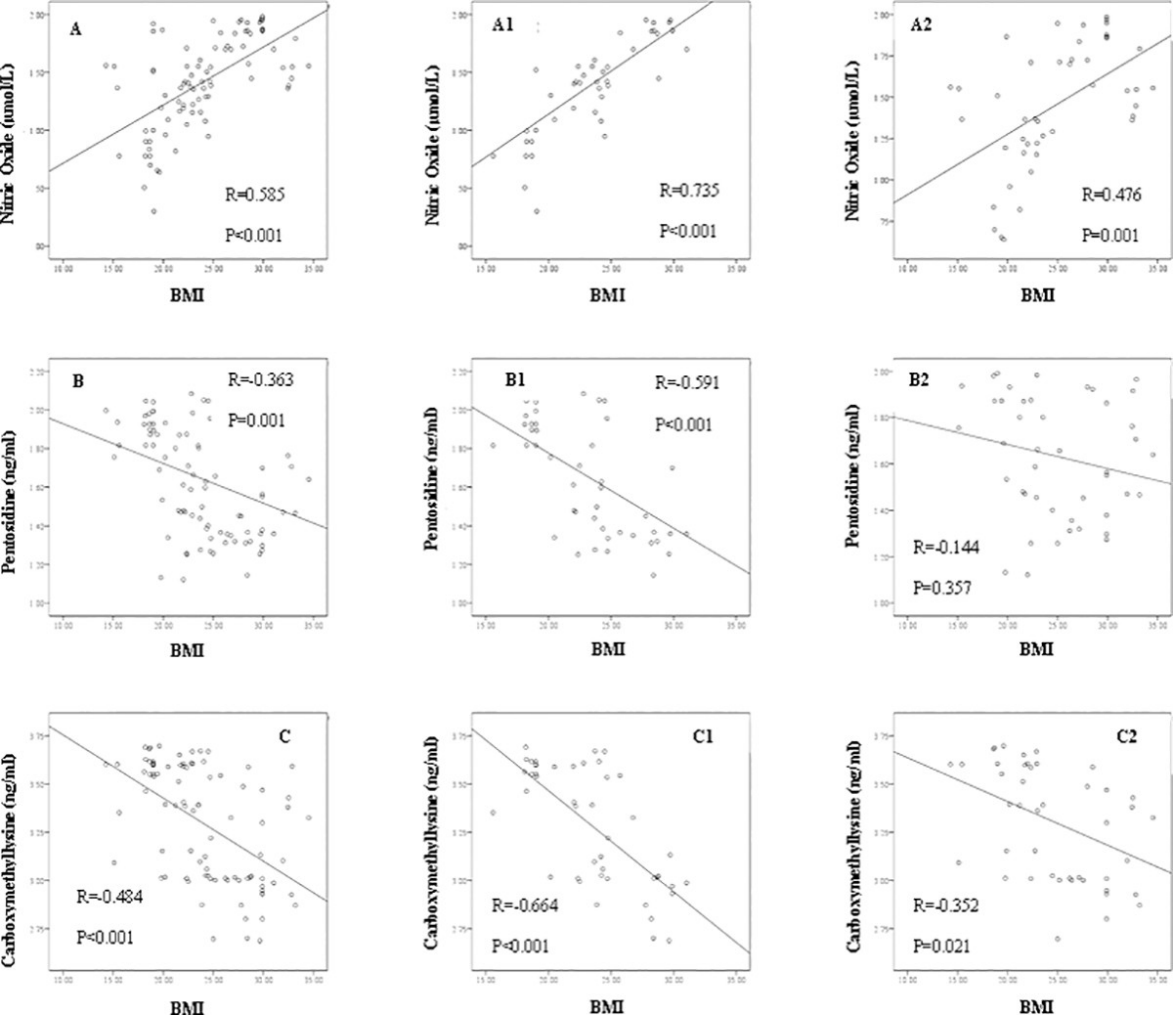
Correlations between serum nitric oxide, pentosidine and carboxymethyllysine with BMI in the total participants (A, B, and C), and based on the gender (A1, B1, and B2 for males), (A2, B2, and C2 for females). P and R are based on the Partial analysis using log-transformed NO, pentosidine, and CML. The confounder effects of age, sex, and WHR were adjusted for A, B, and C and the confounder effects of age and WHR considered for other scatters.

There were no significant differences in serum NO, pentosidine, or CML levels between males and females (Fig 2). Further, a strong positive correlation was determined between serum NO levels and BMI in the subjects (r = 0.585, p<0.001), while serum pentosidine and CML concentrations had negative correlations with BMI (r = -0.363, p = 0.001 and r = -0.484, p<0.001, respectively), after modifying the effects of age, sex, and WHR as confounders (Fig 3). As seen in Fig 3, there was a strong correlations in BMI of male group for NO (r = 0.735, p<0.001), pentosidine (r = -0.591, p<0.001), and CML (r = -0.664, p<0.001). In female group, there were significant correlations between BMI with NO (r = 0.476, p = 0.001) and CML (r = -0.352, p = 0.021), but not pentosidine (r = -0.144, p = 0.357).

Nitric oxide correlated significantly with pentosidine and CML in total participants (r = -0.285, p = 0.008, and r = -0.420, p<0.001, respectively), after adjustment for the effects of age, BMI, WHR and sex (Table 2). With further examined the effect of gender difference in the observed correlation between NO and selected AGE.

**Table 2:** Correlation of nitric oxide (NO) with pentosidine and carboxymethyllysine (CML).

**NO**	**Groups**		**Pentosidine**	**CML**
	BMI≤19.5*	r	0.329	0.001
		p	0.183	0.996
	19.5≤BMI≤24.9*	r	-0.138	**-0.495**
		p	0.453	**0.004**
	BMI≥25*	r	**-0.540**	**-0.377**
		p	**0.002**	**0.037**
	Male**	r	-0.062	-0.123
		p	0.697	0.439
	Female**	r	**-0.372**	**-0.557**
		p	**0.015**	**<0.001**
	Total***	r	**-0.319**	**-0.433**
		p	**0.003**	**<0.001**

*The confounder effects of WHR, sex, and age were adjusted.

**The confounder effects of WHR, BMI, and age were adjusted.

***The confounder effects of WHR, BMI, sex, and age were adjusted.

Based on the Partial correlation

## Discussion

In this cross-sectional study, we measured the serum levels of NO, pentosidine and CML with regard to BMI in apparently healthy subjects. The major findings of the present study are (1) serum levels of NO strongly correlated with BMI, which is raised by increasing BMI, (2) the serum levels of pentosidine and CML inversely associated with BMI, as they were higher in subjects with lower BMI, and (3) serum NO levels is inversely correlated with levels of pentosidine and CML.

It was found that serum NO levels were positively correlated with BMI in both male and female groups. Increased serum NO levels in overweight subjects might be a reflection of increased NO production. In line with our results, animal based studies have shown that plasma and aorta NO levels were significantly higher in obese animals compared to normal weight animals [25, 26]. Several human studies also found that obese and overweight subjects had relatively higher NO levels as compared with normal weight people [27–30]. In Vitale *et al*. study, there was a positive correlation between BMI and salivary NO concentrations in overweight and obese subjects [31]. Choi studied on 319 apparently healthy adolescents and reported that BMI was positively associated with NO in both male and female subjects, as mean BMI in male adolescents with NO>92.8 μmol/l was 26.8± 3.4, which was significantly higher than males with NO<15.6 whose average BMI was 19.9± 4.0 [32]. A recent study has shown that expression of genes which encoding eNOS, iNOS, and cGMP-dependent protein kinase-1 raised in obese women [33]. It has been suggested that eNOS and iNOS are present in white adipose tissue. Obesity induced pro-inflammatory status may upregulate NOS expression [34, 35]. Therefore, the higher presence of NOS in adipose tissue may be a potential source of NO production. Further studies are required to examine if, and to what extent, fat cells are able to release NO.

In this study, lower serum levels of pentosidine and CML were seen in subjects with higher BMI. Gaens *et al* reported that plasma CML was inversely associated with central obesity [36]. Semba *et al* also relieved that subjects with higher CML had lower BMI [37]. Moreover, there are reports that serum soluble receptors of AGE (sRAGE) are inversely correlated with BMI [38, 39]. Taken all together, it has been suggested that excess accumulation of visceral and peripheral adipose tissue in obese subjects may play a role in trapping of AGEs and have a capacity to drive lower serum AGEs. In addition, Lower levels of these products in serum may serve as a marker of greater accumulation/trapping of AGEs in the adipose tissue, where it contributes to the proinflammatory processes associated with obesity. This would promote inflammation in adipose tissue and hence upregulation of iNOS and production of NO. Thus, reductions in circulating AGEs may simply reflect increased uptake into adipose tissue.

We did not notice any gender differences in NO levels of our study participants. In line with our study, Tehrani *et al*. also investigated that serum NO levels of middle-aged subjects had no significant difference between women and men [40]. In an animal based study, Safari *et al*. had not shown any significant difference in the serum NO level between male and female rats [41]. Further, in the population based study of Ghasemi *et al*. on 3505 healthy adults, although serum NO levels peaked between the ages of 50–59 years, but there was not any significant gender difference [42]. In contradictory of our study, a recent study on isolated rat aortae had shown that endothelium derived NO levels in females were significantly more than male rats [43]. In another study, there were no gender difference in urinary NO excretion, but eNOS concentration in whole kidney were 80% higher in female rats than in males [44]. Takahashi *et al*. have shown that plasma NO levels were lower in female compared with males [45]. In Taylor *et al*. study, NO levels in male group was approximately 25% less than females [46]. Overall, some of the contradicted studies reported possible confounding factors including age, lipids, and glucose profile, which could minimize the mentioned gender difference. Further investigation is warranted to enhance our understanding of gender difference in NO generation.

We also have seen no gender differences in serum levels of pentosidine and CML. Ghidoni *et al*. have not reported any significant correlations between RAGEs levels and gender in patients with mild cognitive impairment or control groups [47]. Van Deemter *et al*. conducted a study to investigated associated factors with pentosidine accumulation in the human vitreous. In this study, the authors had not found any correlation between pentosidine and gender [48]. In return, Wang *et al*. have reported that male rats exhibited higher AGEs and oxidative stress compared to female rats [49]. Further, Kilhod *et al*. investigated significantly higher serum levels of AGEs in men than in women in their population based study on 1141 non-diabetic individuals [50]. Further studies are needed to elucidate whether the accumulation of AGEs is contributed to the gender.

Serum levels of NO negatively correlated with CML and pentosidine in study participants. Liao *et al* reported that NO production markedly decreased in the presence of AGEs [51]. Bucala *et al*, in an in vitro study, found that AGEs could inversely modulate NO activity [52]. It has been shown that NO donors including S-Nitroso-N-acetylpenicillamine (SNAP) and sodium nitroprusside (SNP) inhibited AGE-induced hypertrophic growth and receptor for AGE (RAGE) expression through induction of the NO/cGMP/PKG signaling pathway [51]. AGEs also significantly suppressed the NO/cGMP/PKG signaling in human renal proximal tubular cells [53]. Thus, it is speculated that reduction in serum NO are contributed to the higher AGEs concentrations through the mentioned mechanisms.

### Limitations

This study had some limitations. As the numbers of study subjects with BMI above 30 was low (n = 8), it was difficult to make a firm conclusion that serum NO levels in obese individuals is exceptionally higher in obese patients as compared to other study BMI groups. Further, serum NO levels are affected by differences in dietary intake and renal clearance, which were not measured in this study.

## Conclusions

It is concluded that serum NO levels were positively correlated with BMI in both male and female participants. Further, higher BMI contributed to attenuation of serum pentosidine and CML levels. Gender did not affect the variables. Serum levels of NO inversely associated with glycated products. More studies are needed to investigate the mechanisms involved in this relationship.

## Supporting information

S1 Dataset (SAV)
